# Patient involvement in interprofessional education: A qualitative study yielding recommendations on incorporating the patient’s perspective

**DOI:** 10.1111/hex.13073

**Published:** 2020-06-04

**Authors:** Sjim Romme, Matthijs H. Bosveld, Marloes A. Van Bokhoven, Jascha De Nooijer, Hélène Van den Besselaar, Jerôme J. J. Van Dongen

**Affiliations:** ^1^ Maastricht University Maastricht The Netherlands; ^2^ Department of Family Medicine Care and Public Health Research Institute (CAPHRI) Maastricht University Maastricht The Netherlands; ^3^ School of Health Professions Education Maastricht University Maastricht The Netherlands; ^4^ Research Centre for Community Care Zuyd University of Applied Sciences Heerlen The Netherlands

**Keywords:** health professions education, interprofessional education, medical education, patient involvement, patient‐centred care, person‐centred care

## Abstract

**Background:**

Patient involvement in interprofessional education (IPE) is a new approach in fostering person‐centeredness and collaborative competencies in undergraduate students. We developed the Patient As a Person (PAP‐)module to facilitate students in learning from experts by experience (EBEs) living with chronic conditions, in an interprofessional setting. This study aimed to explore the experiences of undergraduate students, EBEs and facilitators with the PAP‐module and formulate recommendations on the design and organization of patient involvement in IPE.

**Methods:**

We collected data from students, EBEs and facilitators, through eight semi‐structured focus group interviews and two individual interviews (N = 51). The interviews took place at Maastricht University, Zuyd University of Applied Sciences and Regional Training Center Leeuwenborgh. Conventional content analysis revealed key themes.

**Results:**

Students reported that learning from EBEs in an interprofessional setting yielded a more comprehensive approach and made them empathize with EBEs. Facilitators found it challenging to address multiple demands from students from different backgrounds and diverse EBEs. EBEs were motivated to improve the person‐centredness of health care and welcomed a renewed sense of purpose.

**Conclusions:**

This study yielded six recommendations: (a) students *from various disciplines* visit an EBE to foster a comprehensive approach, (b) groups of *at least two* students visit EBEs, (c) students may need *aftercare* for which facilitators should be receptive, (d) EBEs need *clear instruction* on their roles, (e) multiple EBEs in one session create *diversity in perspectives* and (f) *training programmes and peer‐to‐peer sessions for facilitators* help them to interact with diverse students and EBEs.

## 1 INTRODUCTION

Increasing prevalence of chronic diseases due to ageing urges western health‐care systems to change.^1^, ^2^ About 35% of the European population is diagnosed with a chronic illness, and this number is expected to increase even further.^3^, ^4^ This rise leads to a need for more clinical integration, implying coordinating person‐centred care in a single process across time, place and discipline.^5^, ^6^, ^7^ Clinical integration requires a person‐centred focus as opposed to solely focusing on the clinical problem.^7^, ^8^ Additionally, patients should be viewed as co‐creators of the care process, who share responsibility with the health‐care professional.^9^, ^10^, ^11^ A systematic review of the effectiveness of person‐centred care suggests a positive relation with care outcomes.^12^

The delivery of integrated person‐centred care requires interprofessional collaboration competencies from health‐care professionals. The quality of interprofessional collaboration in health care is positively related to patient satisfaction and safety.^13^, ^14^, ^15^, ^16^ To foster interprofessional collaboration, professionals should be aware of their role, understand professional boundaries and communicate effectively with colleagues with adjacent backgrounds.^15^, ^17^ Such competencies can be learned via interprofessional education (IPE) training sessions.^18^ IPE is known to help students acquire a more positive attitude towards and appreciation of other professions and their collaborative knowledge and skills.^19^

IPE can be designed using paper cases, simulated patients or by involving real patients, hereafter called experts by experience (EBEs). Students find encounters with EBEs instructive because their authenticity contextualizes students’ learning.^20^ Additionally, students had more positive assumptions and attitudes towards people with chronic illness as a result of active patient involvement.^21^ This outcome is imperative, as this counters a narrow medical focus and subsequently empathy decline in students from various health professions as a result of the hidden curriculum.^22^, ^23^, ^24^ The hidden curriculum comprises of the norms and values that are implicitly transmitted to future health‐care professionals and undermine the formal messages of the curriculum.^25^, ^26^

In addition to students, EBEs also reported positive outcomes of participating in health professions education.^27^, ^28^ They described being pleased to give something back to the community by sharing their experiences and reported increased self‐esteem and empowerment, in‐depth insight into the doctor‐patient relationship and their problems.^27^, ^28^

Although involving EBEs in IPE seems to be a promising approach, the number of examples in literature is limited, and guidelines for its design and implementation are lacking.^29^, ^30^ Predominantly, in existing programmes, single EBEs share their experiences with chronic illness with students, either in a single interview or in a series of encounters.^30^, ^31^

In order to learn to collaborate interprofessionally and to obtain insight in the various roles such as partner, parent, employee and friend, that people fulfil in addition to being a person with a chronic illness, we developed an educational module: Patient As a Person (PAP). The module aims to increase students’ insight into (a) the impact of illness on mental and social dimensions of health and (b) other health‐care disciplines. The PAP‐module consists of three meetings, in which students from various health professions interact with EBEs (Box 1). For a detailed description of students’ various study programmes, see Appendix 1.

### Box 1. Format of Patient as a Person (PAP‐)module

The PAP‐module was developed by students (SR and MB). Students from various disciplines and educational institutions within the health‐care domain (ie a university, a university of applied sciences and two secondary vocational education institutions) interacted with people with chronic conditions in three meetings. A PAP‐group consisted of five EBEs and ten students from various educational institutions. Practically, this means that roughly two‐thirds of students followed the module at another educational institution than their own. Diversity in student backgrounds was strived for when composing every PAP‐group, to the extent that scheduling constraints allowed. An overview of the participants’ backgrounds can be found in Appendix 1.

EBEs were asked to share their views on health and health care with students, using their experiences to illustrate potential improvements in health‐care delivery. These experiences could be painful and intimate. Confidentiality was assured by referring to students’ professional behaviour. EBEs were recruited via primary care practices, hospitals and patient associations. Teachers acted as facilitators of the group process and will, therefore, be referred to as facilitators. Their tasks included safeguarding a confidential and encouraging environment in which both students and EBEs dared to be vulnerable, moderating the group conversation and structuring the meetings. Facilitators were present during the plenary first and third meeting only.

**I: Plenary meeting at educational institution focusing on the health‐care system**

A PAP‐group has a plenary meeting, conducted in a half‐circle with the facilitator at the open end. An introduction aims at creating an atmosphere of trust. The person‐centeredness and current state of interprofessional collaboration in health care are discussed. EBEs are encouraged to attend the session together with an informal caregiver. 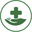


**II: Pairs of students visit an EBE and discuss their life with a chronic condition**

Two students with different educational backgrounds visit one EBE. These student pairs are composed to create diversity in backgrounds. Pairs, rather than larger groups, realize an atmosphere of trust and confidentiality. The EBE shares how disease has impacted his or her mental and social well‐being. Moreover, the EBE reflects whether and how health‐care professionals have addressed these dimensions adequately and provides suggestions for future improvements in professional‐patient communication. Students can, but are not obliged to, use a framework with various dimensions of health to structure their conversation (Appendix 2).^32^ 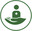


**Assessment**

After visiting the EBE, students write a reflective essay in which they elaborate on (a) how they will cater to patients’ needs concerning the mental and social impact of health in their future work and (b) whether (and if so, what) they learned from the perspective of the student of another profession. Additionally, students highlight similarities and differences between their perspectives and those of their fellow student.

**III: Plenary meeting at educational institution focusing on synthesizing learning outcomes**

The translation of lessons learned into future practice is the central theme in this final session. All five groups of two students share the lessons they learned in Meeting II and discuss these with fellow students and the EBEs. Commonalities and differences between the five stories are discussed to broaden the learning experience. 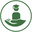


2

Other than existing programmes in which a single EBE interacts with students, PAP‐groups include five EBEs with various disease‐ and demographic backgrounds, to create diversity in the experiences and opinions of EBEs.^28^ EBEs receive a short practical instruction before the PAP‐module, in order to guarantee the authenticity of their experiences. The purpose of this study is to explore the experiences of all stakeholders involved (undergraduate students, experts by experience and facilitators) with the PAP‐module in order to formulate recommendations for the optimal design and organization of patient involvement in IPE.

## 3 METHODS

### 3.1 Study design

Using a qualitative study design, we explored the individual experiences of all stakeholders involved. Focus group interviews and individual interviews were conducted between March 2018 and May 2019. We interviewed EBEs, students and facilitators separately so they could talk freely about their roles and the roles of other stakeholders. Our theoretical orientation was based on social constructivism which uses the premise that social interaction leads to the development of new insights.^33^ This way, researchers can explore hidden assumptions.^34^, ^35^ We report relevant aspects of this study using the Consolidated Criteria for Reporting Qualitative Research (COREQ).^36^, ^37^ This study was approved by the Netherlands Association for Medical Education (NVMO) Ethical Review Board: NERB dossier number 992.

### 3.2 Setting and participants

In the PAP‐module, 216 students from three institutions participated: Maastricht University (health sciences and medicine, first year), Zuyd University of Applied Sciences (speech therapy, physiotherapy and nursing, third and fourth year) and Regional Training Center Leeuwenborgh (home care provision, third and fourth year). An explanation of students' backgrounds, as well as an overview of the number of participants of the PAP‐module, can be found in Appendix 1.

We conducted eight semi‐structured focus group interviews and two semi‐structured qualitative interviews with a total of 51 participants (Table 1). We conducted four focus group interviews and two individual interviews with students, two focus group interviews with EBEs and two with facilitators.

**Table 1:** Participant characteristics

Students				
ID	Gender	Age	Study programme	Educational institution
SU1	Female	18‐22	Health Sciences	Maastricht University
SU2	Female	18‐22	Health Sciences	Maastricht University
SU3	Female	18‐22	Health Sciences	Maastricht University
SU4	Female	18‐22	Medicine	Maastricht University
SU5	Female	18‐22	Medicine	Maastricht University
SU6	Female	18‐22	Health Sciences	Maastricht University
SU7	Female	18‐22	Health Sciences	Maastricht University
SU8	Male	18‐22	Health Sciences	Maastricht University
SU9	Male	18‐22	Medicine	Maastricht University
SU10	Female	23‐27	Medicine	Maastricht University
SU11	Male	23‐27	Medicine	Maastricht University
SU12	Female	18‐22	Medicine	Maastricht University
SU13	Male	18‐22	Medicine	Maastricht University
SU14	Female	18‐22	Medicine	Maastricht University
SU15	Female	18‐22	Medicine	Maastricht University
SZ1	Female	23‐27	Speech therapy	Zuyd University of Applied Sciences
SZ2	Female	18‐22	Speech therapy	Zuyd University of Applied Sciences
SV1	Female	18‐22	Home care provision	ROC Leeuwenborgh (secondary vocational education)
SV2	Female	18‐22	Home care provision	ROC Leeuwenborgh (secondary vocational education)
SV3	Female	18‐22	Home care provision	ROC Leeuwenborgh (secondary vocational education)
SV4	Female	18‐22	Home care provision	ROC Leeuwenborgh (secondary vocational education)
SV5	Female	18‐22	Home care provision	ROC Leeuwenborgh (secondary vocational education)
SV6	Female	18‐22	Home care provision	ROC Leeuwenborgh (secondary vocational education)
SV7	Female	18‐22	Home care provision	ROC Leeuwenborgh (secondary vocational education)
SV8	Female	18‐22	Home care provision	ROC Leeuwenborgh (secondary vocational education)
SV9	Female	18‐22	Home care provision	ROC Leeuwenborgh (secondary vocational education)
Experts by experience				
ID	Gender	Age	Role	Educational institution
EBE1	Male	58‐62	EBE	Maastricht University
EBE2	Male	63‐66	EBE	Maastricht University
EBE3	Female	50‐54	EBE	Maastricht University
EBE4	Male	58‐62	EBE	Maastricht University
EBE5	Female	58‐62	EBE	Maastricht University
EBE6	Male	63‐66	Informal caregiver	Maastricht University
EBE7	Male	63‐66	EBE	Maastricht University
EBE8	Female	63‐66	EBE	Maastricht University
EBE9	Male	67‐71	EBE	Zuyd University of Applied Sciences
EBE10	Female	58‐62	EBE	Zuyd University of Applied Sciences
EBE11	Female	63‐66	EBE	Zuyd University of Applied Sciences
EBE12	Female	50‐54	EBE	Zuyd University of Applied Sciences
EBE13	Male	23‐27	EBE	Zuyd University of Applied Sciences
EBE14	Male	67‐71	Informal caregiver	Zuyd University of Applied Sciences
EBE15	Male	67‐71	EBE	Zuyd University of Applied Sciences
EBE16	Male	50‐54	EBE	Zuyd University of Applied Sciences
Facilitators				
ID	Gender	Age	Educational institution	Department
FU1	Female	30‐39	Maastricht University	Health Education and Promotion
FU2	Female	50‐59	Maastricht University	Skillslab
FU3	Female	60‐69	Maastricht University	Health Ethics and Society
FU4	Female	20‐29	Maastricht University	Freelancer
FU5	Female	40‐49	Maastricht University	Skillslab
FZ1	Male	50‐59	Zuyd University of Applied Sciences	Research centre of community care
FZ2	Female	40‐49	Zuyd University of Applied Sciences	Speech therapy
FZ3	Male	30‐39	Zuyd University of Applied Sciences	Research centre of community care
FZ4	Female	30‐39	Zuyd University of Applied Sciences	Speech therapy
FZ5	Female	40‐49	Zuyd University of Applied Sciences	Nursing
FZ6	Female	40‐49	Zuyd University of Applied Sciences	Speech Therapy

The main inclusion criterion for participation in the study was having followed the entire PAP‐module. The EBEs who participated in this study had a diverse range of illnesses including acquired brain injury, amputation, post‐traumatic stress disorder, cancer, neurological and psychiatric illness. To recruit EBEs, we used purposive sampling, aiming to obtain diversity in terms of disease background, age and gender. The facilitators asked EBEs face‐to‐face whether they would like to participate in the study. We contacted those EBEs willing to participate, to plan the interview. We selected facilitators and students using convenience sampling and recruited them via e‐mail after they completed the PAP‐module. Non‐responding among students from Zuyd University was primarily attributable to students being at internships and having examinations. EBEs received reimbursement for their travel expenses, students received a ten euro gift card, and facilitators received no reward. We informed all participants about the goal of the interviews using written information provided two weeks in advance and verbal explanation just before the interviews. An informed consent form was signed when they agreed.

### 3.3 Data collection

All interviews took place in quiet rooms. The focus group interviews lasted approximately 100 minutes, whereas the individual interviews lasted 45 minutes. During all interviews, a semi‐structured interview guide was used by the moderator (Appendix 3). Moderators had experience with moderating focus group interviews and had backgrounds in communication skills education, interprofessional learning communities and qualitative research. The interviews were audio‐taped, transcribed verbatim, summarized and participants were asked to provide feedback on this summary as a member check, which two participants did. An anonymized version of the data on which this study is based is available upon request. The data were encrypted, and the key was stored on a separate server.

### 3.4 Data analysis

We used conventional content analysis to analyse the transcripts.^38^ The transcripts of each source (ie students, EBEs and facilitators) were separately and independently analysed by SR, MB and JvDo using open coding. Next, using axial coding, we individually grouped the relevant codes into subcategories.^34^ Afterwards, we compared subcategories until we reached consensus and categorized them into key categories (Appendix 4). The preliminary results section, including an extensive set of quotes, was subsequently discussed with JdN and LvB. We used Nvivo 12 to organize and code the data.^39^

## 4 RESULTS

### 4.1 Students

#### 4.1.1 Insight into the impact of illness on mental and social dimensions of health

Students expressed that the PAP‐module made them empathize more with EBEs. For example, asking for consent to start a treatment or examination felt unnatural to students during consultation practice. After hearing EBEs’ stories, students better understood their desire for autonomy and control in a professional‐patient relationship. Health sciences students reported that the PAP‐module made EBE preferences more tangible, which deepened their understanding of the policy perspective of their study programme. Medicine students explained that they were more curious and empathized more with the EBE when compared to encounters with simulated patients. Additionally, they stated that in their consultation training, developing an understanding of the medical problem seems to be the goal. In the PAP‐module, they had the opportunity to explore how the person's life changed as a result of illness.

Student University of Applied Sciences 2: “You really learn how to empathize. In our education, we learn about all diseases and their respective treatments, but we do not learn what is accompanying this illness, what is going on in the person. That is what I learned in this module, that is very important”.

Students reported that interacting with multiple EBEs showed them that preferences regarding interacting with health‐care professionals are heterogeneous. For example, some EBEs preferred only factual information, whereas others preferred extensive personal attention. These different preferences made them realize that there is no general best way of displaying empathy and comforting EBEs. Lastly, students reported having gained an understanding of the EBEs’ hardship, like coping with shrunken social networks, worsened financial situations and a life without the possibility of paid work. Students were impressed by the EBEs’ often positive attitudes towards life regardless of their severe hardship.

Student University of Applied Sciences 1: “The scope was broader than it is normally (in IPE activities). We focused more on the context. Often, we ask, what is the problem? Family is briefly addressed; wife, children sometimes, but we do not delve into that”.

Furthermore, they realized that EBEs’ dependency on care did not stop after hospital discharge. They mentioned that aftercare and social care should be personalized to an EBE's needs and personal environment. Students reported that interacting with “EBEs as persons”, including their roles as partners, parents, employees *as well as* their role as patients, led to increased attention for aspects of their context, such as family and social network, professional background and living environment. Furthermore, they had not realized the importance of this context until after the module.

Student University 10: “Here at university, we learn about [CANMEDS] competencies such as health advocate. We learn that you have to consider the patients’ home environment. For me, before participating in PAP, these were empty words. I thought it was obvious. This [module] has shown me that paying attention to this is really important. It provided meaning”.

#### 4.1.2 Interprofessional learning

Students reported that the module made them more aware that care should be delivered in an integrated way and should cross boundaries of professions. They stated that when care is delivered in an empathic yet fragmented way, EBEs will not experience it as person‐centred. Students said that they had limited initial knowledge about the roles of other health professions. They learned more about other professions involved in the PAP‐module. Occupational therapists and physiotherapists became aware of their differing foci, “participation in society” and “activities”, respectively. Consequently, they asked different questions which led to different degrees of attention for the EBEs’ context. Similarly, medicine students realized that they had a more narrow focus when compared to nursing and occupational therapy students.

Student University 13: “I noticed that they [occupational therapy students] asked very different questions… At a certain moment, I noticed that I followed their approach. Initially, I asked questions like: how did you get ill and which medication do you use? Yet, they asked questions like: how do you shop for groceries now and do you sleep upstairs or downstairs? That's quite important, whether someone can actually climb the stairs in their home”.

Additionally, students from both universities reported that the approach common in their fields was more formal and analytical compared to the approach they observed in the students from the vocational level. These students from the universities stated that a more intuitive approach might enable them to connect with EBEs’ needs better.

Students reported that they found the interprofessional collaboration inspiring because they saw how students from different disciplines were passionate about their future work. Health sciences students found it very interesting to get more insight into the practicalities of daily work life of health‐care professionals in terms of (time)pressure and hierarchy on the work floor. Students from the vocational level reported having gained a more positive view of their profession and increased self‐esteem, especially when compared to their more theoretically educated peers.

Student University 7: “….We, as health scientists, can talk about making consultations longer so that space emerges for doctor's empathy, so they have time to ask follow‐up questions. I think patients miss that and doctors do not have time”.

Student Secondary Vocational Education 3: “I always looked up to doctors and nurses, but actually I have more experience in talking with patients than they do… That gave me a good feeling. So, I have always had a too low perception of myself”.

### 4.2 Experts by experience

#### 4.2.1 Reasons for participating

A primary reason for EBEs to participate was improving communication in health care by contributing to the development of students. EBEs stressed that training in empathy and collaborative competencies could help prevent some of their negative experiences from occurring again with future EBEs.

EBE 8: “They (doctors) are robots. They have much knowledge, but that is useless if you do not have empathy. I see it as my task, as an EBE, to train the doctor”.

EBE 10: “I am reinventing myself after what happened. I search for a sense of purpose and a new routine, and that is one of the reasons I am sitting here now. I want to share my experiences, my mission and contribute for other people”.

Additionally, EBEs found it valuable to target stigmata since they experienced prejudice from society and said that people look down on them.

EBE 4: “I fully enjoy telling (students) that patients should not be pitied but are capable of many things. Only in a different manner”.

#### 4.2.2 Assertiveness and recognition

EBEs reported having become more assertive in professional‐patient relationships and that they reflected on their general behaviour and attitudes.

EBE 4: “We are talking a lot about the student and what he learns from all this, but I have to say it was helpful to me too. … During the third meeting, I noticed, based on my responses, that I had become quite a negative person. … In such a meeting with students, this is emphasized. That was a personal learning outcome.”

EBE 7: “So yeah, you become more aware…: hey, as a patient, I can fulfil a role in the interaction with health care professionals”.

Some EBEs reported that sharing their experiences made them feel heard, which they believed to have a therapeutic effect.

EBE 6: “What I find quite important, I might not be a patient (but a caregiver instead), but I noticed it is tough to find a place where you can share your story. You cannot do that at the doctor, because you only have ten minutes. You can just put it on the table here, everybody does it, so it has a therapeutic effect”.

EBEs reported that the PAP‐module lowered the threshold for them to open up, meet other EBEs and participate in society again, which was regarded as desirable since their social circles became smaller due to illness.

EBE 11: “For us, participating in this project is coming back into social life. If you have lived between your bed and the chair for twelve years, not much is left of your social life”.

### 4.3 Facilitators

#### 4.3.1 Dealing with multiple demands

Facilitators described moderating the plenary meetings as very demanding. They found it ambitious to combine the intended learning outcomes related to IPE with seeing the patient as a whole in one short module. Demanding aspects included managing time and adjusting to the frames of reference and communication styles of EBEs and students with different backgrounds and educational levels, especially since most facilitators only had experience in teaching one discipline. However, the majority of the facilitators reported that they found it rewarding to engage with students and EBEs in a deep and personal way.

Facilitator University of Applied Sciences 3: “It is demanding in terms of guiding the process, guarding time and content… you want students to learn from each other so the interprofessional aspect should be there. You want to improve the learning process by asking the right questions, and you want to involve EBEs at the same time”.

Furthermore, facilitators found it demanding to guide EBEs in telling their stories to students in a constructive way without EBEs focusing too much on venting their experiences.

Facilitator University of Applied Sciences 1: “This can be a double agenda too. On the one hand, the EBE has a therapeutic goal, and you (as a facilitator) have an educational goal. That can clash a bit sometimes”.

Facilitator University 6: “The EBE is in the lead quite a lot. They think, well ok, this is my moment. When the student said something afterwards, they would add some things immediately. This led to the student talking a bit less”.

### 4.4 Design

#### 4.4.1 Fostering an encouraging learning atmosphere

Facilitators, EBEs and students reported that it was essential to foster an encouraging learning atmosphere in which everyone felt valued and dared to be vulnerable. After all, EBEs were asked to share personal and sometimes painful experiences of living with chronic illness in plenary meetings. Facilitators disclosed personal experiences to display personal vulnerability in order to create an atmosphere of trust and equality. Some facilitators used this as a specific intervention, whereas others described this as something that happened spontaneously. One facilitator expressed being unwilling to share personal experiences because of privacy considerations.

Facilitator University of Applied Sciences 3: “This works as an intervention. By acting vulnerable in the beginning, you create a safe environment that makes the next person feel comfortable to share something personal too”.

Facilitator University of Applied Sciences 2: ‘For me, it is a matter of emotion. I did not use it consciously’.

#### 4.4.2 Sensitivity to students' vulnerability

Facilitators reported that some students were particularly vulnerable in the PAP‐module because they had experiences with illness in their personal situation. They recommended a delicate approach towards students, mainly since the mental and social components of illness are discussed as part of the module. EBEs knew beforehand that sharing personal information is their task in the module. However, for some students, the stories of EBEs made them relive their experiences with health care as a patient (relative). Students sometimes shared these stories, which they might not have expected beforehand. Facilitators said that students should receive information about the possibility of reliving painful personal experiences and that the possibility of aftercare for students should be present. EBEs confirmed this by stating that they sometimes felt that it was intense for the students to process EBEs’ experiences.

Facilitator University of Applied Sciences 1: “I noticed that some students were in a double role. On the one hand, as a student, on the other hand, they had past experiences as a patient. At some point, this played a role in the discussion. This was enriching, but, … it complicated the session”.

EBE 11: “We share quite a lot with those young folks. Sometimes we think, auch, this might be a bit too much?”

### 4.5 Recruitment

Students reported that EBEs should be able to reflect on their experiences with illness and care while transcending their emotions to be able to contribute to students' development adequately. Other students reported that they would prefer no screening on EBEs’ ability to transcend their emotions because this does not represent the reality of their future working life. Furthermore, students preferred EBEs’ stories to be authentic and their backgrounds diverse. Students reported that it is essential that EBEs have a critical attitude towards the care they experienced while being open to students’ contributions. Some students noted that their EBEs were very negative about health‐care professionals and believed the EBEs extrapolated this negativity towards them.

Student University 11: “I think it is important to ask the EBEs to approach the module with an open mind‐set. Because I think… At PAP, there are quite some negative people with extensive knowledge about health care. When they sit at the other side of the table, they can be quite sceptical about the encounter with us. They have to be open to the process and understand that we are still students”.

Furthermore, facilitators and students reported that they believed that participating EBEs were more verbally skilled and able to cope with life with a chronic disease than most EBEs. They thought that these EBEs were more experienced and invested more in improving health care than most EBEs. EBEs confirmed that less proactive EBEs are less likely to participate in the PAP‐module.

In some groups, informal caregivers participated along with the EBEs. Facilitators and students reported that these different perspectives were insightful. EBEs confirmed this: they regard informal caregivers as having equivalent experience with the health‐care system, which added a new perspective to the EBE's experience.

### 4.6 Participant preparation

Some EBEs misunderstood students’ genuine interest in their stories as a sign that the students were interested in friendly contact outside the confined meetings of the PAP‐module. One student reported being glad to visit EBEs in their home together with a fellow student. Her fellow took it upon her to introduce the end of the encounter. This helped her to realize her discomfort with having to finalize the encounter with the EBE. She became aware that her compassion with the EBE compromised her professional boundaries. Facilitators separately volunteered that more students experienced such difficulties.

Some EBEs reported that students sometimes gave limited feedback on whether the PAP‐experience was helpful, which was disappointing for them. Additionally, some EBEs had doubts about the long‐term effects of their contributions to students’ behaviours.

EBE 1: “They listen to your story and during the session they say "yeah, it had quite an impact," but you do not really get insight in what this means for their future”.

## 5 DISCUSSION

### 5.1 Summary of findings

This study aimed to explore the experiences of undergraduate students, experts by experience (EBEs) and facilitators with the Patient As a Person (PAP‐)module. Talking with EBEs about the social and mental domains of health in a non‐medical setting made students more aware of the need for a person‐centred approach. Additionally, students realized that they need the input of other professions to realize this. For EBEs, participation in the PAP‐module was advantageous as well, because they felt heard, it increased their assertiveness, and it gave them a sense of purpose. However, some had doubts about the lasting effect of the module on the students' professional behaviour. Some EBEs misinterpreted students’ interest in them as a sign of companionship that would last after the PAP‐module had concluded. For facilitators, it was challenging to deal with multiple demands, such as being sensitive to the emotional strain on students, interacting with students from different professional backgrounds as well as diverse EBEs in one group.

### 5.2 Conclusions on involving EBEs into IPE

Based on the experiences of students, EBEs and facilitators, we formulated recommendations regarding the design and organization of IPE involving EBEs.

#### 5.2.1 Interprofessional learning in the EBEs’ home context

Meeting EBEs in their home context and talking with the EBE about various domains of health, together with students from different disciplines, made students adopt a more comprehensive approach. The interprofessional learning of students primarily takes place in either classrooms or professional practice. In contrast, EBEs experience the consequences of their illnesses mostly in their home environments.^40^ Our study shows that the home context of the EBE can be an appropriate place to start learning about the social and mental impact of illness. This approach could make students look at EBEs as individuals who experienced a change in the roles they fulfil in life as a result of illness and help them in coping with this change. This form of education might be instrumental in complementing the hidden curriculum, which encompasses the implicit and often unintended learning of students and includes a narrow medical focus and consequently, empathy decline.^25^, ^26^ By learning to see the EBE’s perspective, students learn about the importance of empathy which could counter some implicitly learned values such as detachment and distrust of emotions.^23^, ^25^, ^26^

The different approaches towards EBEs that students from different professions have, were accentuated by talking with an EBE in pairs. Previous studies show that IPE using simulated patients increases students’ teamwork and communication skills.^20^, ^41^ However, while simulation can be instrumental in enhancing communication skills, it is argued that simulation lacks authenticity and makes students act empathically without learning them to establish a genuine connection with people.^42^ Students in our study found it valuable to identify how students with different backgrounds made different connections with EBEs.

Recommendation 1: Let students from various disciplines visit EBEs in their home context together to make them adopt a more comprehensive approach and identify similarities and differences with other professions.

#### 5.2.2 Aftercare for students

Our study indicates that being receptive to students in need of aftercare is imperative as they might relive painful, personal experiences in health care, and they can have a double role as a student and patient (relative). Facilitators should be receptive, and students should know that there is an opportunity to share painful experiences with a confidentiality advisor. Reflecting on such difficult interactions and coping with them is a part of professional identity formation, which refers to the moral and professional development of students and the integration of students’ individual maturation and their growth in professional competency.^43^, ^44^ Coping with such emotions can be seen as a form of professional identity formation which, when mentored appropriately, can add to health‐care professional resilience.^43^, ^44^

In addition to aftercare, EBEs should be made aware of the temporary nature of the contacts as students sometimes felt discomfort in ending the contact, because they felt pressure to keep in touch with EBEs who experienced loneliness. On the other hand, students should be made aware of the need to be clear about their professional boundaries, as clear professional boundaries can prevent compassion fatigue in health‐care professionals.^45^ To lower students' threshold in ending the home visit, all meetings that take place in the EBEs’ home context should be attended by at least two students.

Recommendation 2: Let students visit EBEs in pairs or larger groups.

Recommendation 3: Be receptive to students needing aftercare as a result of reliving painful personal experiences in health care as a patient (relative).

#### 5.2.3 Training of experts by experience

Providing only a practical instruction, yet no training to EBEs, yielded authentic EBE experiences which made students empathize with EBEs. However, in some instances, students and facilitators believed that EBEs focused more on venting their experiences with a therapeutic goal for themselves as opposed to constructively sharing their experiences to help students. Our study highlights the importance of clearly instructing EBEs on the intended learning outcomes of the module. The extent to which EBEs need extensive training is debated in the literature and varies considerably between different programmes involving EBEs as teachers.^46^, ^47^ Training about strategies in facilitating interprofessional learning tends to decrease EBEs’ perceived authenticity.^46^ When the authenticity of EBEs' contributions is essential, as it is in our programme, training and extensive selection of EBEs might be undesired. However, this could have the consequence of including some EBEs who focus primarily on venting their own experiences.

Recommendation 4: Instruct EBEs about their roles and responsibilities, including the temporary nature of the contact with students.

#### 5.2.4 Involving multiple EBEs and caregivers

Our results revealed that involving multiple EBEs in one PAP‐group with multiple students showed two advantages. First, EBEs had diverse opinions, needs and preferences, which made students more aware of the uniqueness of every EBE. This supports findings by Towle and Godolphin, who state that panel discussions, including multiple EBEs, create a diversity of EBEs’ perspectives and therefore stimulate balanced learning.^28^ Second, EBEs described that participating with other EBEs in the confined environment of the PAP‐module lowered their thresholds to open up. Furthermore, they said that meeting other EBEs led to peer support. Becoming chronically ill often leads to losing a substantial part of a person's social network.^48^, ^49^ This can lead to social isolation, characterized by feelings of marginality, exclusion, loneliness and hopelessness, which have detrimental health consequences.^50^ Hence, facilitating EBEs in expanding their networks can yield health benefits for EBEs via stronger social ties and can increase their satisfaction with participating in health professions education.

Recommendation 5: Involve multiple EBEs in one session to create diversity in perspectives and to lower the thresholds for EBEs to open up and provide and receive peers support.

#### 5.2.5 Training of facilitators in IPE

Facilitators often had experience in one specific health profession and therefore found it difficult to connect with the frames of reference of students of other health professions. Previous studies confirm that facilitators experience IPE as complex and demanding to lead due to the variety of students and learning needs.^51^ Regular peer‐to‐peer sessions with facilitators from different disciplines have been proposed to overcome this complexity as it enables facilitators to share their uncertainties and learn from and about different disciplines.^51^ An additional issue was facilitators’ uncertainty in dealing with EBEs, who differed considerably in terms of their focus on contributing to students’ learning or venting their own experiences. While we could not find examples of interventions for this issue in the literature, our personal experience was that instruction sessions, including authentic role‐play with EBEs enhanced facilitators’ skills and self‐esteem in leading a session involving EBEs.

Recommendation 6: Organise instruction‐ and peer‐to‐peer sessions for facilitators focusing on dealing with students from different disciplines and diverse EBEs.

### 5.3 Strengths and limitations

Our study had a wide mixture of participants. We included students and facilitators from different disciplines, educational levels and institutions and EBEs with different illnesses. This increased the richness of the data.

However, the results of our study cannot simply be extrapolated to other circumstances. Some limitations must be acknowledged. Despite the mixture of participants involved, we were able to include only two students from the university of applied sciences level, whom both studied speech therapy. Therefore, students’ views on IPE with students from different levels are predominantly based on the opinions of students from university and vocational level. Perhaps students from the university of applied sciences level had different experiences with the PAP‐module. Hence, data saturation was not achieved for students from all educational levels.

Additionally, the EBE population that participates in the PAP‐module is not a representative sample of people with chronic illness because they were more verbally skilled and invested in improving health care. However, some ability of the participants to introspect and verbalize seems to be essential for the success of this programme. EBEs in the PAP‐module participate voluntarily, which means that the EBEs have time and are willing to contribute to health professions education and are mobile enough to travel. This resulted in an overrepresentation of EBEs of 50 to 70 years old.

Another limitation is the double role fulfilled by SR and MB. They initiated and developed the module and analysed the interviews as researchers. Therefore, they were at risk of having a biased view of the results. To tackle this potential limitation, the interviews were moderated by independent researchers and the transcripts were coded by a third, independent, researcher (JvDo). The preliminary results section, with an extensive set of quotes, was subsequently discussed with three other researchers (LvB, JdN and HvB) who were not involved in the PAP‐module.

## 6 CONCLUSION

This studies’ results are extrapolated in several recommendations for the design and organization of interprofessional education involving real patients, that is experts by experience (EBEs). Learning with students from various professions and educational levels in the EBEs home context results in a positive attitude towards a more comprehensive approach and provides them with insight into other disciplines. Additionally, being receptive of students who need aftercare is essential. Students should visit EBEs in pairs or larger groups and clearly instructing EBEs and students about the goals of the encounters is essential. Furthermore, involving multiple EBEs and caregivers in one session creates diversity in EBE perspectives which leads to a valuable exchange and lowers the thresholds for EBEs to open up. Lastly, facilitators need appropriate training in terms of dealing with students from different disciplines as well as with diverse EBEs. Further research on the transfer of lessons learned in the context of the EBE to daily professional practice is necessary, to make claims about behavioural changes in students.

## CONFLICT OF INTEREST

At the time of conduct of the study, Romme and Bosveld were in the board of the Patient As a Person Foundation, the organization that developed the educational module. The other authors declare no conflicts of interest concerning the authorship or the publication of this article.

## ETHICAL REVIEW BOARD

Approval for conducting this study was received from the ethical review board from the Dutch Association for Medical Education (NVMO): NERB dossier number 992.

## Data Availability

An anonymized version of the data on which this study is based is available upon request.

## Appendix 1

### PARTICIPANTS OF THE PAP‐MODULE

In total, 216 students participated, of which 108 were first‐year university‐level students (medicine or health sciences) from Maastricht University, 51 were third‐ or fourth‐year students in speech therapy, physiotherapy, and nursing of Zuyd University of Applied Sciences, and 57 were students from Vista College or Gilde Opleidingen, two institutes for vocational education in the Netherlands (Table 1). EBEs (N = 86) suffered from a diverse range of diseases such as acquired brain injury, respiratory disease, chronic cancer, multiple sclerosis and psychological illnesses. Teachers could sign‐up for PAP voluntarily at Maastricht University, but were assigned by management at Zuyd University of Applied Sciences and the vocational institutions.

**Table A1:** Participants in PAP‐training

Participant	Number of participants	Study programme (for students)
Students		
University	108	Medicine or health sciences (first year)^a^
University of Applied Sciences	51	Speech therapy, physiotherapy, nursing (third and fourth year)
Secondary vocational education	57	Home care provision (third and fourth year)^b^
EBEs	86^c^	
Teachers	24^d^	

^a^ Health sciences are a three‐year bachelor's programme that focuses on the relations between health, behaviour, nutrition, environment, social environment and health care. Graduates do not obtain a professional registration.

^b^ Home care provision is a three‐year study on the secondary vocational level. Graduates receive a professional registration, and their responsibilities include dressing up patients, washing them and providing basic medication. In the Netherlands, three levels of tertiary education are distinguished: secondary vocational education, university of applied sciences and university (ordered from practice‐oriented to theory‐oriented). In this study, one institution of all three levels was included, and PAP‐groups contained students from all three educational institutions.

^c^ 86 EBEs participated 108 times with a range of one to three times per person.

^d^ Some teachers led the groups in pairs, whereas others led them alone.

## Appendix 3

### SEMI‐STRUCTURED INTERVIEW GUIDE

**Students**

1. Open introduction:
  - What did you like about the PAP‐module? What are areas for improvement?
    ▪ Organisational matters
    ▪ Content‐related matters
  - What was the most important take‐away for you?
2. Comprehensive approach/empathy:
  - Do you believe the PAP‐module helped you in adopting a comprehensive approach towards EBEs? If so, could you give an example? If not, why?
3. Involving EBEs:
  - Do you believe involving EBEs in your education has added value? If so, what would this added value be? If not, why?
  - Which traits should participating EBEs possess?
4. Attitude towards interprofessional education:
  - Do you think it is useful to include students from different professions *within* the university/university of applied sciences?
  - Do you think it is useful to include students from different professions *from different levels of education*?

**Facilitators**

1. Open introduction:
  - What did you like about the PAP‐module? What are areas for improvement?
    ▪ Organisational matters
    ▪ Content‐related matters
  - What was the most important take‐away for you?
2. Attitude towards interprofessional education:
  - Do you think it is useful to include students from different professions *within* the university/university of applied sciences?
  - Do you think it is useful to include students from different professions *from different levels of education*?
3. Do you think that the university/university of applied sciences should involve EBEs more systematically in the curricula of health professions?

**EBEs**

1. Open introduction:
  - What did you like about the PAP‐module? What are areas for improvement?
    ▪ Organisational matters
    ▪ Content‐related matters
  - What was the most important take‐away for you?
2. Would you participate again? If so, why? If not, why?
3. What did you like about interacting with students? What was less pleasant?
